# Development of a Framework for the Investigation of Speed, Power, and Kinematic Patterns in Para Cross-Country Sit-Skiing: A Case Study of an LW12 Athlete

**DOI:** 10.3389/fspor.2019.00004

**Published:** 2019-07-31

**Authors:** Julia Kathrin Baumgart, Pål Haugnes, Lars Morten Bardal, Sindre Østerås, Jan Kocbach, Øyvind Sandbakk

**Affiliations:** ^1^Department of Neuromedicine and Movement Science, Faculty of Medicine and Health Sciences, Centre for Elite Sports Research, Norwegian University of Science and Technology, Trondheim, Norway; ^2^Department of Civil and Environmental Engineering, Faculty of Engineering, Centre for Sports Facilities and Technology, Norwegian University of Science and Technology, Trondheim, Norway

**Keywords:** disability, GPS, GNSS, Paralympic, race course analysis, XC skiing

## Abstract

**Objective:** To develop a framework for the investigation of speed, power, and kinematic patterns across varying terrain in cross-country (XC) sit-skiing, and to test this framework in a XC sit-skier of the LW12 class during high- (HIT) and low-intensity (LIT) endurance training.

**Methods:** One XC sit-skiing athlete of the LW12 class with a single above-the-knee amputation was equipped with a GNSS enabled sports watch with integrated barometry and heart rate monitoring (peak heart rate: 195 beats·min^−1^), and an inertial measurement unit. After a warm-up, he performed two 20-m maximal speed tests on a flat and an uphill section to determine maximal speed and power, followed by skiing 5.75 km at both LIT and HIT in varying terrain.

**Results:** 51, 28, and 21% of the time during HIT and 53, 28, and 19% of the time during LIT were spent in uphill, flat and downhill terrain, respectively. Maximal speed in the uphill and flat section was 4.0 and 6.2 m·s^−1^, respectively, and the corresponding maximal power output 342 and 252 W. The % of maximal speed did not differ between the uphill and the flat section (HIT: 66 vs. 67%, LIT: 47 vs. 50%), whereas the % of maximal power output was lower in the uphill than flat section (HIT: 65 and 80%, LIT: 46 and 58%). Still, the absolute power output was slightly higher in the uphill than the flat section (HIT: 222 vs. 201 W, LIT: 156 vs. 145 W). Furthermore, cycle rate was significantly higher during HIT than LIT (60–61 vs. 45–55 cycles·min^−1^, across all terrains, all *p* < 0.03), while cycle length was longer in the uphill terrain (3.0 vs. 2.6 m, *p* < 0.001). Furthermore, the % of peak heart rate was significantly higher in HIT than LIT (90 vs. 78, 85 vs. 67, and 88 vs. 66%, respectively, in the uphill, flat and downhill terrain, all *p* < 0.001).

**Conclusions:** Here, we present a new integrative framework for future investigations of performance, technical and physical demands in XC sit-skiing. In this case study, the increase in speed from LIT to HIT was due to increases in cycle rate in all terrains, while cycle length was less affected. Although the absolute power output was slightly higher in the uphill compared to the flat section both for HIT and LIT, the athlete worked closer to his maximum power output in the flat section.

## Introduction

Cross-country (XC) sit-skiing is performed by athletes with impairments of the lower extremities and/or trunk, who use the upper-body double poling (UBP) technique while sitting in a sledge mounted on two XC skis. Since XC sit-skiing competitions range from ~1 to 15 km and race courses typically consist of undulating terrain with uphill, flat and downhill sections, their training should also reflect these conditions. Accordingly, most of the training performed by XC sit-skiers is upper-body endurance training at low-intensity (LIT), interspersed by high-intensity training (HIT) 1–3 times each week to develop competition-specific technique and related physiological capacities.

All propulsion during training and competition in XC sit-skiing is done by UBP (Gastaldi et al., 2012), which is in contrast to able-bodied cross-country skiing where different whole-body exercise sub-techniques are employed in response to the varying terrain (Nilsson et al., 2004). Despite this difference, the most performance determining sections in XC sit-skiing are the uphill sections (Bernardi et al., 2013), which is in line with able-bodied XC skiing (Sandbakk et al., 2011; Bolger et al., 2015; Haugnes et al., 2019). Furthermore, a significant reduction in speed over the race has been observed in XC sit-skiing competitions, with accompanying reductions in cycle length both during an uphill and a flat section (Bernardi et al., 2013). However, current research is limited to analyses of specific sections where solely video is used to analyse XC sit-skiing (Gastaldi et al., 2012; Bernardi et al., 2013). To better understand overall performance, as well as the physical and technical demands in XC sit-skiing, continuous measurements of speed, and the corresponding power output, kinematic patterns (i.e., cycle rate and length) and heart rate (HR), as a proxy of metabolic intensity, for the various terrains throughout training and competitions would provide important information. In this context, the utilization of combined Global Navigation Satellite Systems (GNSS) and micro-sensor technology in the field has shown potential in able-bodied XC skiers (Sandbakk et al., 2011, 2016; Marsland et al., 2012, 2015, 2017, 2018; Seeberg et al., 2017; Solli et al., 2018) and could provide valuable insight also in XC sit-skiers.

In order to reduce the effect of disability on the performance outcome of a competition, XC sit-skiers are divided into five different classes (LW10-LW12, from the most to the least disabled with the LW12 class being the reference class), with each class being attributed a time factor to determine the finishing rank (Nordic Skiing Classification, 2017). However, the effect of the course profile on this time factor is not taken into account, and currently the possible effects of terrain and conditions on performance in athletes with different disabilities are unknown. Such information is vital both for understanding the performance demands in XC sit-skiing and to provide sport-specific insight into the classification system of XC sit-skiers.

In this case study, the main aim was to develop an integrative framework for the investigation of performance, technical and physical demands in XC sit-skiing. Specifically, we combined GNSS and micro-sensor technology to investigate fluctuations in speed, power, and kinematic patterns during race pace (i.e., HIT) and LIT across varying terrain in a XC sit-ski athlete of the LW12 class.

## Materials and Methods

### Participant

The participant was a male elite Norwegian Para XC sit-skier with a single above-the-knee amputation (age: 45–50 years, height: 165–170 cm, body mass + mass of the sit-ski/poles/skis: 65–70 kg + 2.2/0.7/1.3 kg, peak oxygen uptake (VO_2peak_): 52.8 mL·kg^−1^·min^−1^, peak heart rate (HR_peak_): 195 beats·min^−1^, 1-RM pull-down strength: 115 kg, competition class: LW12). We decided to use a XC sit-skier of the LW12 class in this case study, since this is the reference class for the XC sit-skiing category. This study was pre-approved by the Norwegian Centre for Research Data (ID 49865/3/IJJ) and performed according to the declarations of Helsinki. Prior to the data collection, the participant provided written informed consent to voluntarily take part in the study and to publishing his identifiable data, case description and identifiable images. The participant was informed that he could withdraw from the study at any point in time without providing a reason for doing so.

### Overall Design

The testing consisted of three test days: On day one, the athlete was tested for aerodynamic drag in the wind tunnel. On day two, he performed a ski-snow friction test, as well as two 20-m maximal speed (V_max_) tests both in flat and uphill terrain, followed by 5.75 km (i.e., three laps of 1.92 km) at both LIT and HIT in varying terrain. Here, power output was calculated as the sum of power against gravity (P_g_), ski-snow friction (P_f_), and aerodynamic drag (P_d_) in an uphill and a flat section during the V_max_ tests, and in the same two sections during LIT and HIT. On day three, the athlete performed an incremental test to exhaustion to determine VO_2peak_ and peak HR, as well as a 1-RM pull-down strength test, and had a DXA scan taken.

### Test Protocols and Instruments

#### Wind Tunnel Testing

**Figure 1:**
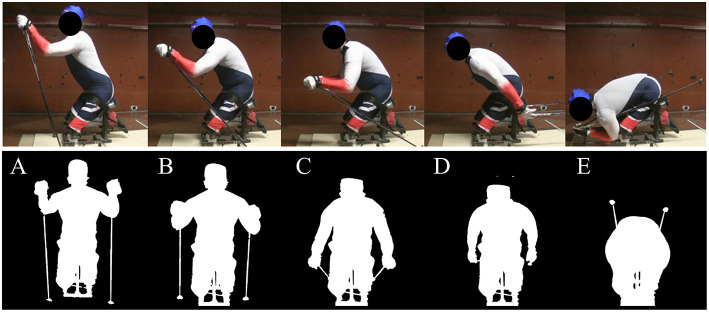
The skier's positions and frontal area during an upper-body poling movement cycle from high **(A)** to mid-high **(B)**, mid-low **(C)**, and low **(D)**, as well as the tuck position **(E)** used in the downhill sections. The participant provided written informed consent to publishing the identifiable images in this figure.

The aerodynamic drag for various parts of the skier's full movement cycle in the UBP technique and for the tuck position was measured in the wind tunnel of the Norwegian University of Science and Technology (Figure 1). The sit-ski was fixed to a six-component strain-gauge force platform, and the mean drag force was measured over 20 s at four different velocities, between 15 and 50 km/h, in each position. The skier's projected frontal area was estimated from pictures using pixel counting. The calculated area was used to correct the wind tunnel measurement for blockage effects experienced in a closed test-section wind tunnel.

#### Outdoor Testing

##### V_*max*_ tests

**Figure 2:**
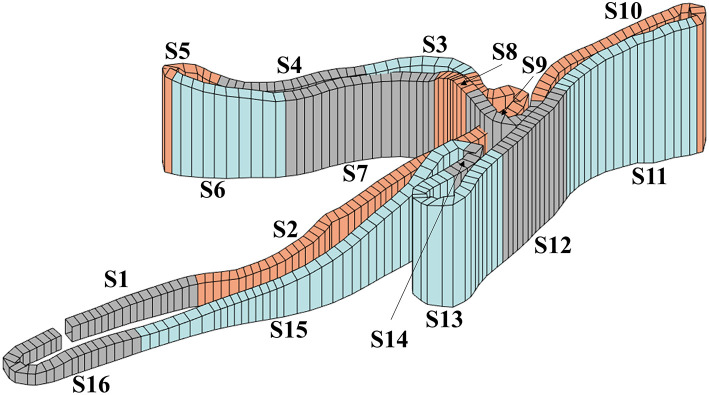
Race course with uphill sections in orange, flat sections in gray and downhill sections in blue. One lap consisted of four uphill sections (S2, S5, S8, and S10) with mean inclines of 8, 14, 16, and 10% and section lengths of 348, 54, 33, and 157 m, seven flat sections (S1, S4, S7, S9, S12, S14, and S16) with section length of 91, 112, 117, 90, 82, 38, and 151, and five downhill sections (S3, S6, S11, S13, and S15) with mean slopes of −9, −10, −7, −6, and −10% and section length of 56, 76, 113, 128, and 269 m.

The sit-skier used his own ski equipment, including the sit-ski, poles and skis, which were prepared according to the prevailing conditions. Prior to testing, the sit-skier warmed up for 10 min. He then performed two 20-m V_max_ tests with a self-selected run-up in the first flat section (Figure 2), followed by two 20-m V_max_ tests in the first following uphill section (Figure 2) as described by Solli et al. (2018).

##### Low- and high-intensity training

A 10-min recovery period with easy skiing followed the V_max_ tests before the XC sit-skier performed two 5.75 km on a competition track, one time at LIT (rate of perceived exertion (RPE) 8–14) and one time at HIT (i.e., race pace). During skiing, GNSS data, altitude, and HR were recorded with a Garmin Forerunner 920XT (Garmin Ltd., Olathe, KS, USA) with sampling rate of 1 Hz as described in more detail by Sandbakk et al. (2016) and video was recorded continuously with a Garmin VIRB camera (Garmin Ltd, Olathe, KS, USA) mounted frontally on the head of the skier and pointing downwards. In one lap of HIT we lost GNSS signal during part of the race course, and used GNSS data from the GNSS receiver integrated in the Garmin VIRB camera instead. After the two 5.75 km, RPE was recorded and a blood sample taken to measure blood lactate concentration (BLa) with a Biosen C-Line Sport lactate measurement system (EKF-diagnostic GmbH, Magdeburg, Germany). Five min of light activity was performed between the two intensities. The snow and weather conditions were relatively stable throughout the testing with light wind, a partly cloudy sky, wet snow, an air temperature of −0.5°C, a snow temperature of −0.7°C, ~90% humidity and an atmospheric pressure of ~955.0 hPa. The track was packed with hard-packed mixed snow and machine-prepared in the morning prior to testing. The total course length was 5,754 m (3 × 1,918 m) with varied topography based on a course profile (Figure 2) divided into uphill, flat, and downhill terrain that made up 31, 35, and 34% of the track, respectively.

##### Ski-snow friction tests

Prior to LIT and after HIT, the skier performed three ski-snow friction tests in each direction on the 20-m section used for the flat V_max_ tests. The skier had an initial speed of ~5.5 m·s^−1^ when entering the 20-m flat measurement zone and then passively glided while sitting in a tucked down position (Sandbakk et al., 2011). The ski-snow friction was measured as 0.039 before the LIT activity and 0.033 after the HIT-activity, respectively, ignoring the force of air drag which was minimal at this slow speed. The mean value (0.036) was used for all power calculations.

##### Section time, speed, and power calculations

The time spent in each section was calculated based on virtual split times. Mean speed for each section was calculated by dividing the length of a track section by the time elapsed within this terrain section. The track length was measured using the same GNSS as used for tracking the athlete. Expected accuracy of the distance measurement using this type of GNSS is around 2–4%. In accordance with the results in Gløersen et al. (2018), we did not use the distance covered by the GNSS receiver for time analysis purposes in the current study, because differences in the length trajectories per lap then accumulate over time. Instead, following the recommendations in Gløersen et al. (2018), we used a common mapping trajectory in the time analysis. This gives a section time error of 0.4–0.9 s for 20–180-m long sections, with the error in section time plateauing for longer sections. The mean power output for the uphill and the flat section was calculated as the sum of power (*P*_*tot*_*)* against gravity (*P*_*g*_), snow friction (*P*_f_), and aerodynamic drag (*P*_*d*_), with v being the mean speed, ∝ the angle of incline, μ_s_ the ski-snow friction coefficient, ρ the density of the air, *A* the exposed frontal area of the skier, and *C*_d_ the aerodynamic drag coefficient (Equations 1, 2).

(1) $$\begin{array}{l} P_{\mathrm{tot}}=P_{g}+P_{f}+P_{d} \end{array}$$

(2) $$\begin{array}{l} P_{\mathrm{tot}}=\text{m }\cdot \text{g }\cdot \sin\left(\propto \right)\cdot v+m\;\cdot g\;\cdot cos\left(\propto \right)\cdot \;\mu_{s}\;\cdot v\\ \;+0.5\cdot \rho \;\cdot v^{3}\;\cdot A\;\cdot C_{d} \end{array}$$

The aerodynamic drag area (*A* · *C*_*d*_) was 0.338 m^2^ (mean of position mid-high (B) and mid-low (C) of Figure 1) for the uphill and 0.336 m^2^ for the flat section (mean of position high (A) to low (D) of Figure 1). The density of air (*p*) was set to 1.29 kg/m^3^.

##### Algorithm for UBP cycle rate and length calculation

**Figure 3:**
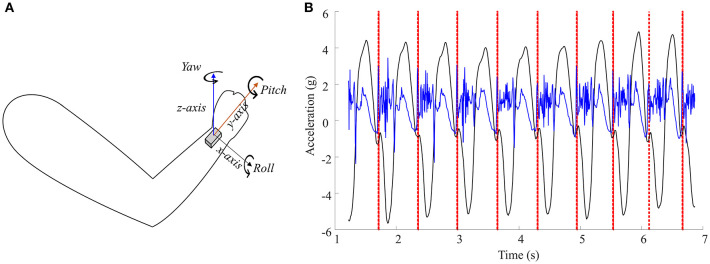
**(A)** Illustration of the axes of the inertial measurement unit attached to the skier's right wrist. **(B)** Visualization of the algorithm. Pole plants are found by first identifying peaks in the x-component (roll angle) of the gyroscope-data (black line) corresponding to maximum angular speed when the skier raises the arm, and then by finding the next spike in the z-component of the accelerometer-data (blue line) corresponding to the following impact of the right pole (red dotted lines).

Cycle rate was determined from each pole plant, as defined by an algorithm utilizing a combination of the accelerometer and gyroscope data from the inertial measurement unit (IMU (Physilog®5, Gaitup, Switzerland) (see Figure 3). The algorithm was validated using video recordings with the VIRB camera in one HIT lap, resulting in a success rate of >95%. Misclassifications of the algorithm were related to making turns or movements that deviated from the double poling movement, such as stretching the arm. Cycle time was calculated as the time between pole plants, and cycles accepted as an UBP cycle when the time between pole plants ranged between 0.6 and 3 s. Time differences between pole plants exceeding 3 s can be attributed to the skier sitting in the tucked down position or making a turn, and were not counted as an UBP cycle. Cycle rate was calculated based on cycle time as cycles·min^−1^. Cycle length was calculated based on the distance covered between pole plants by combining the cycle time with the mean speed from GNSS data. The accuracy of the GNSS allows us to calculate mean speed and cycle length averaged over each section with sufficient precision (Gløersen et al., 2018). However, calculation of cycle length for single cycles requires a more accurate GNSS, which is planned in a follow-up study using a similar approach.

#### Laboratory Testing

##### VO_**2peak**_ test

The sit-skier performed the incremental test in his own competition XC sit-ski, which was firmly fixed to a wooden platform in front of the Concept2 ski ergometer (Concept2, Morrisville. VT. USA). The equipment used to assess body mass, as well as respiratory parameters, BLa and RPE during the incremental test in accordance with Baumgart et al. (2018b). HR was assessed with the same Garmin Forerunner 920XT used during the outdoor testing.

##### Maximal strength test

The procedure and equipment used to assess pull-down 1-RM strength were in accordance with the shoulder extension exercise performed in Østerås et al. (2016). A 10-min warm-up was performed on a ski ergometer (Concept2 Inc., Morrisville, VT, USA).

#### Statistics

The XC sit-skier performed three laps during both HIT and LIT. Each lap consisted of four uphill sections (S2, S5, S8, and S10), seven flat sections (S1, S4, S7, S9, S12, S14, and S16), and five downhill sections (S3, S6, S11, S13, and S15) (detailed metadata contained in Supplementary Table 1). This allowed us to use paired-samples T-tests in SPSS 22.0 (Software for Windows, SPSS Inc., Chicago, IL, USA) to compare speed, cycle rate and length, and % of peak heart rate in the different terrains and between HIT and LIT. Furthermore, independent-samples T-tests were used to compare the latter variables between the different terrains. An alpha level of 0.05 was used to indicate statistical significance. However, the reader should be aware that the generalizability of the analyses remains limited due to the investigation of only one participant.

## Results

We were able to successfully use the integrative framework developed in this case study to investigate fluctuations in speed, power, and kinematic patterns during race pace (i.e., HIT) and LIT across varying terrain in a XC sit-ski athlete of the LW12 class.

The XC sit-skier spent 1,340 s during HIT (430, 454, and 456 s during lap 1, 2, and 3, respectively) and was 27% slower with 1,829 s during LIT (576, 640, and 613 s during lap 1, 2, and 3, respectively) to complete the 5.75 km course. The overall RPE was 19 during HIT (19, 19, and 14 for the uphill, flat and downhill terrain, respectively) and 14 during LIT (16, 12, and 9 for the uphill, flat and downhill terrain, respectively), with a corresponding % of HR_peak_ of 88 and 70%, respectively, during HIT and LIT. Furthermore, the blood lactate concentration was 11.5 mmol·L^−1^ after HIT and 2.7 mmol·L^−1^ after LIT. 51, 28, and 21% of the time during HIT and 53, 28, and 19% of the time during LIT was spent in uphill, flat, and downhill terrain, respectively.

**Figure 4:**
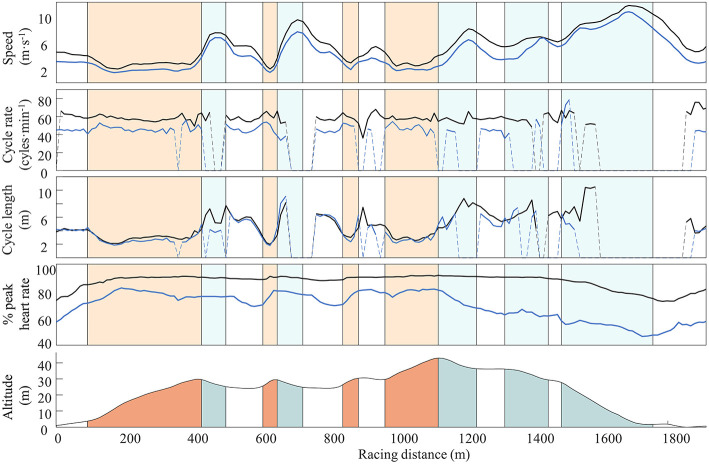
Mean speed, cycle rate, cycle length, heart rate of three laps with high-intensity exercise (black line), and three laps with low-intensity exercise (blue line) over a race course of 1.92 km with varying terrain. The dotted lines show where the sit-skier does not upper-body double pole, i.e., during most of the downhill sections of the course.

Maximal speed was 4.0 and 6.2 m·s^−1^ in the uphill and flat section, respectively, and maximal power output was 342 W (P_g_: 66%, P_f_: 30%, P_d_: 4%) and 252 W (P_g_: 17%, P_f_: 63%, P_d_: 20%), respectively. Table 1 and Figure 4 provide an overview over speed, power output, kinematic variables (i.e., cycle rate and cycle length) and heart rate in the different terrain types of the race course. The % of maximal speed did not differ between the uphill and the flat section neither during HIT (i.e., 1 p.p.) nor LIT (i.e., 3 p.p.). However, the % of maximal power output tended to be lower in the uphill than flat section during HIT (i.e., 15 p.p.) and LIT (i.e., 12 p.p.), whereas the absolute power output values were slightly higher in the uphill compared to the flat section (21-W difference during HIT and 11-W difference during LIT).

**Table 1:** Mean speed, cycle rate, cycle length and % of peak heart rate (±SD_pooled_) in uphill, flat and downhill terrain and % of maximal speed, power output and % of maximal power output (PO) (±SD) in a defined uphill and flat section in each of the three laps during HIT and LIT.

	**Speed (m/s)**			**% of max speed**		**Power output (W)**		**% of max PO (W)**		**Cycle rate (cycles·min**^****−1****^**)**			**Cycle length (m)**			**% of peak heart rate**		
	**Uphill**	**Flat**	**Down**	**Uphill**	**Flat**	**Uphill**	**Flat**	**Uphill**	**Flat**	**Uphill**	**Flat**	**Down**	**Uphill**	**Flat**	**Down**	**Uphill**	**Flat**	**Down**
HIT1	2.5 ± 0.3	5.9 ± 1.0	6.6 ± 1.6	71	75	239	231	70	92	61 ± 2	60 ± 3	62 ± 6	3.0 ± 0.4	5.8 ± 1.1	6.4 ± 1.0	88 ± 1	83 ± 9	88 ± 2
HIT2	2.3 ± 0.5	5.8 ± 1.2	7.5 ± 2.0	63	64	212	192	62	76	59 ± 2	60 ± 4	60 ± 7	2.8 ± 0.2	5.3 ± 0.7	6.6 ± 0.6	90 ± 1	85 ± 5	88 ± 4
HIT3	2.4 ± 0.4	5.3 ± 1.0	7.1 ± 1.6	64	61	215	181	63	72	58 ± 2	61 ± 6	62 ± 7	3.1 ± 0.5	5.2 ± 0.7	6.3 ± 0.9	92 ± 1	87 ± 6	89 ± 5
Overall mean ± SD	2.4 ± 0.4	5.7 ± 1.1	7.1 ± 1.7	66 ± 4	67 ± 8	222 ± 15	201 ± 26	65 ± 4	80 ± 10	60 ± 2	60 ± 5	61 ± 7	3.0 ± 0.4	5.4 ± 0.9	6.4 ± 0.8	90 ± 1	85 ± 7	88 ± 4
LIT1	1.8 ± 0.3	4.3 ± 0.7	5.8 ± 1.3	51	53	170	157	50	62	50 ± 2	46 ± 2	56 ± 16	2.8 ± 0.3	5.5 ± 0.8	5.8 ± 0.6	79 ± 3	68 ± 8	70 ± 9
LIT2	1.6 ± 0.2	4.1 ± 1.3	5.8 ± 1.5	43	44	143	127	42	50	49 ± 2	44 ± 1	51 ± 17	2.5 ± 0.2	5.3 ± 1.4	6.1 ± 1.9	77 ± 2	66 ± 9	65 ± 12
LIT3	1.7 ± 0.2	4.2 ± 1.0	5.6 ± 1.7	47	52	156	152	46	60	49 ± 2	46 ± 3	57 ± 18	2.6 ± 0.2	5.3 ± 0.8	5.3 ± 1.0	77 ± 2	66 ± 9	64 ± 12
Overall mean ± SD	1.7 ± 0.3	4.2 ± 1.0	5.7 ± 1.5	47 ± 4	50 ± 5	156 ± 13	145 ± 16	46 ± 4	58 ± 6	49 ± 2	45 ± 2	55 ± 17	2.6 ± 0.2	5.4 ± 1.0	5.7 ± 1.3	78 ± 2	67 ± 9	66 ± 11

Cycle rate was significantly higher for HIT than LIT in the uphill, flat and downhill terrain (all *p* < 0.03). In contrast, cycle length was not different between HIT and LIT in the flat and downhill terrain (both *p* > 0.13), but slightly longer in the uphill terrain (*p* < 0.001). Furthermore, % of peak heart rate was significantly higher in HIT than LIT in the uphill, flat, and downhill terrain (all *p* < 0.001).

## Discussion

This is the first study to provide a framework for the investigation of performance, technical and physical demands in XC sit-skiing. More specifically, we combined GNSS and micro-sensor technology to investigate speed, power, and kinematic patterns at race pace (i.e., HIT) and during LIT in a XC sit-skier of the LW12 class. Both for HIT and LIT, the XC sit-skier spent most time in uphill terrain where he reached both the highest power output and heart rate. The increase in speed from LIT to HIT was mainly due to increases in cycle rate, while cycle length was longer only in the uphill terrain. Although the absolute values of power output were slightly higher in the uphill compared to the flat section, the XC sit-skier worked closer to his maximum power output in the flat section.

Similar to what has previously been found in able-bodied XC skiers (Bolger et al., 2015; Sandbakk et al., 2016), our Para XC sit-skier spent around 50% of the time in the uphill terrain both for HIT and LIT. Although this was expected, it indicates a high importance of the uphill terrain for overall performance also in Para XC sit-skiing. This finding is substantiated by Bernardi et al. (2013), who showed that the uphill terrain was the main differentiator between low and high-level performers by using video analysis in few sections during a XC sit-skiing competition at the Paralympic games. In this case study, we extended on their findings by continuous measures of GNSS, technique variables and heart rate, which provided insight into performance, physical and technical demands across the entire session in a XC sit-skier of the LW12 class. In the future, the framework provided could be used to investigate to what extent training and performance demands differ for terrain-specific sections between the various XC sit-ski classes.

This approach allowed us to provide new insight into the athlete's pacing strategy and the related kinematic patterns, both for the entire course as well as for each of the sections. During HIT in this study, the XC sit-skier used a positive pacing strategy with lap 2 and 3 being 5–6% slower compared to lap 1. This is in line with a previous study on XC sit-skiers (Bernardi et al., 2013) as well as studies on standing able-bodied XC skiers (Bolger et al., 2015; Sandbakk et al., 2016). However, there was no clear pattern in kinematic changes associated with the loss of speed during these laps, and HR slightly increased over the laps, which indicates that effort was maintained.

Although the performance demands are well-simulated by HIT in the current study, the majority of training is LIT for both XC sit-skiers (unpublished results from the training diaries of the Norwegian XC sit-skiers) and their able-bodied counterparts (Tønnessen et al., 2014; Sandbakk and Holmberg, 2017). Here, the mean speed was 27% lower during LIT compared to HIT, with speed in the uphill, flat and downhill terrain being 29, 26, and 20% lower for LIT compared to HIT. Despite the relatively lower speed in the uphill terrain, the metabolic intensity and effort in the uphill terrain during LIT were closer to HIT (RPE: 16 vs. 19 of HR_peak_: 78 vs. 90%) as compared to the flat and downhill terrain. The relatively high metabolic intensity and effort in our case study in the uphill terrain during LIT can likely be explained by the constraints of uphill XC sit-skiing, where it is beneficial to maintain speed at a level where the system (i.e., sledge and skier) is constantly moving forward without any full stop in the steepest segments. This is more pronounced than in able-bodied XC skiers who are better able to regulate intensity in all terrain types including the uphill terrain during LIT (Haugnes et al., 2019). Overall, our findings highlight the relevance of such information to understand the demands of training and competition.

Due to the constraints of the double poling movement in XC sit-skiing, where the range of motion and time to produce propulsion is limited, the lower speed in LIT is attributed to a lower cycle rate compared to HIT, whereas cycle length showed to be less affected by intensity with a slightly longer cycle length during HIT being present only in the uphill terrain. This is different than what has been found in able-bodied XC skiers where both cycle rate and length are used to increase or reduce speed between such intensities across all terrains (Sandbakk and Holmberg, 2017). However, for double poling in able-bodied XC skiers, speed increases are more related to increases in cycle rate than for other techniques due to the limited time to produce propulsion when poling. This effect might be even more pronounced for XC sit-skiers due to both shorter range of motion during poling and lack of leg-work supporting their upper-body poling, and could explain the observed difference compared to able-bodied XC skiers.

This is the first study to estimate maximal power output for a XC sit-skier during on-snow skiing. The ability to produce 342 W by upper-body poling when XC sit-skiing is similar to the 340 W found in male able-bodied XC skiers by Hegge et al. (2015b) for the same movement during 30-s all-out sprints on a skiergometer. However, XC sit-skiing on snow is more technically demanding and has a faster contraction velocity and shorter poling times compared to using a skiergometer (Rosso et al., 2017). Longer poling times are likely also the reason for the 90 W higher power produced when skiing at maximal speed uphill compared to flat terrain in this case study.

Obviously, standing XC skiers produce higher speed and power output for the same terrain, both during the V_max_ tests as well as during HIT and LIT (Stöggl and Holmberg, 2016; Solli et al., 2018) compared to the XC sit-skier in this case study. This can be attributed to a reduced amount of active muscle mass during XC sit-skiing where only the upper-body is used for propulsion. Even though the VO_2peak_ of the XC sit-skier (53 mL·kg^−1^·min^−1^) in this study is above the mean provided for a group of Nordic sit-skiers in a previous study (46 mL·kg^−1^·min^−1^) (Baumgart et al., 2018a), it is much lower compared to elite standing XC skiers (80–90 mL·kg^−1^·min^−1^) (Haugen et al., 2018). Accordingly, training in the sitting mode taxes the cardio-respiratory system to a lesser extent (Reybrouck et al., 1975; Hegge et al., 2015a; Baumgart et al., 2017). Furthermore, not only the absolute values but also the % of maximal speed and power output utilized during HIT and LIT were lower than what has previously been found in XC skiers (Stöggl and Holmberg, 2016; Solli et al., 2018; Haugnes et al., 2019). Why the relative values are lower in our case is not known, but it might be related to an earlier onset of muscular fatigue with isolated upper-body work compared to whole body work with variations in the use of multiple sub-techniques.

In addition, the % of maximal power output was lower in the uphill compared to the flat terrain (65 vs. 80% during HIT and 46 vs. 58% during LIT), despite a similar % of maximal speed (66–67% during HIT and 47–50% during LIT). This shows that although the XC sit-ski athlete produces higher absolute power uphill, he is still able to work closer to the maximum in the flat compared to the uphill terrain. The latter might be due to the technical limitations in producing the high speeds required to provide high maximal power output in flat terrain during the V_max_ test, which seems to be less limiting during HIT and LIT where longer poling times are achieved due to lower speeds.

HR fluctuated quite extensively during LIT (i.e., higher HR in the uphill, lower HR in the downhill terrain), whereas fluctuations were reduced during HIT. For HIT, HR was nearly unaffected by terrain and was basically the same in uphill and downhill terrains (i.e., there is little HR recovery). This is partly attributed to delayed HR kinetics, where the demands of the heavy uphill sections are also reflected in the subsequent downhills. Although delayed HR kinetics have also been found in able-bodied skiers (Bolger et al., 2015; Solli et al., 2018; Haugnes et al., 2019), the able-bodied skiers seem to recover more in the downhill sections than the XC sit-skier in our study. This might be attributed to slower HR response when using the upper-body isolated when XC sit-skiing in steep uphill terrain, and/or less possibility for recovery in the following downhill sections when steering the sledge at high speeds and in challenging turns. The latter are aspects that need further elucidation, but coaches and XC sit-ski athletes should be aware of these when analysing their competition and training demands.

The methodology of data acquisition and analysis used in this case study will allow us to examine the influence of terrain and conditions on performance in general and across the different Para XC sit-ski classes (LW10-12) in future studies. Therefore, the framework developed here can also be used to investigate how the time factor used for the different Para sit-ski classes varies across terrains and, hence, if an adjustment of the time factor based on the course profile should be considered.

### Methodological Considerations

As with all case studies, the certainty with which we can conclude on our findings remains limited due to only one athlete being tested. Furthermore, the results of this case study cannot be generalized to other XC sit-ski athletes, especially to athletes of the lower classes with a higher level of impairment. In addition, there are limitations in the accuracy of some of the outcome measurements. For example, the variation in the friction coefficient can likely be attributed to differences in skier position and ski-snow contact; factors that are hard to standardize in an outdoor setting. A further example is the 2–4% measurement error associated with the use of a GNSS enabled watch with limited accuracy, from which data was retrieved to calculate the track section length and speed. However, the methodology provided here together with more accurate GPS/GNSS tracking can be applied in future studies to provide insight into the validity of the time factor used in Para XC sit- and standing skiers, as well as novel information about the training and performance demands in Para XC sit-skiing and similar sports.

## Conclusion

In this case study, we present a new integrative framework for future investigations of performance, technical and physical demands in XC sit-skiing. In the XC sit-skier of the LW12 class, the increase in speed from LIT to HIT was due to increases in cycle rate in all terrains, while cycle length during HIT was longer only in the uphill terrain. In the future, combined analyses of speed, power, and kinematic variables with higher accuracy and using experimental designs can aid in understanding the effect of terrain and condition on the time-factors in the classification process of Para athletes and allow unique insights into training and performance demands of Para XC sit-skiers which is of high interest for both coaches and athletes.

## Data Availability

All datasets generated for this study are included in the manuscript and/or the Supplementary Material.

## Ethics Statement

This study was pre-approved by the Norwegian Centre for Research Data (ID 49865/3/IJJ) and performed according to the declarations of Helsinki. Prior to the data collection, the participant provided written informed consent to voluntarily take part in the study and to publishing his identifiable data, case description, and identifiable images. The participant was informed that he could withdraw from the study at any point in time without providing a reason for doing so.

## Author Contributions

JB, PH, LB, SØ, JK, and ØS all contributed to the conceptualization and design of the study. JB, PH, LB, and ØS acquired the data. JB and PH analyzed the data and JB, JK, and ØS interpreted the data. JB drafted the study with all authors critically revising it for important intellectual content. The final version sent in for publication was approved by all authors and all authors agreed to be held accountable for all aspects of the work.

### Conflict of Interest Statement

The authors declare that the research was conducted in the absence of any commercial or financial relationships that could be construed as a potential conflict of interest.
